# Human Cysteine Cathepsins Are Not Reliable Markers of Infection by *Pseudomonas aeruginosa* in Cystic Fibrosis

**DOI:** 10.1371/journal.pone.0025577

**Published:** 2011-09-28

**Authors:** Clément Naudin, Alix Joulin-Giet, Gérard Couetdic, Patrick Plésiat, Aneta Szymanska, Emilia Gorna, Francis Gauthier, Franciszek Kasprzykowski, Fabien Lecaille, Gilles Lalmanach

**Affiliations:** 1 Inserm U618, Université François Rabelais, Protéases et Vectorisation Pulmonaires, Tours, France; 2 Laboratoire de Bactériologie, CHU Jean Minjoz, Besançon, France; 3 Faculty of Chemistry, Department of Medicinal Chemistry, University of Gdansk, Sobieskiego, Gdansk, Poland; Universität Heidelberg, Germany

## Abstract

Cysteine cathepsins have emerged as new players in inflammatory lung disorders. Their activities are dramatically increased in the sputum of cystic fibrosis (CF) patients, suggesting that they are involved in the pathophysiology of CF. We have characterized the cathepsins in CF expectorations and evaluated their use as markers of colonization by *Pseudomonas aeruginosa*. The concentrations of active cathepsins B, H, K, L and S were the same in *P. aeruginosa*-positive (19 Ps+) and *P. aeruginosa*-negative (6 Ps−) samples, unlike those of human neutrophil elastase. Also the cathepsin inhibitory potential and the cathepsins/cathepsin inhibitors imbalance remained unchanged and similar (∼2-fold) in the Ps+ and Ps− groups (p<0.001), which correlated with the breakdown of their circulating cystatin-like inhibitors (kininogens). Procathepsins, which may be activated autocatalytically, are a potential proteolytic reservoir. Immunoblotting and active-site labeling identified the double-chain cathepsin B, the major cathepsin in CF sputum, as the main molecular form in both *Ps+* and *Ps−* samples, despite the possible release of the ∼31 kDa single-chain form from procathepsin B by sputum elastase. Thus, the hydrolytic activity of cysteine cathepsins was not correlated with bacterial colonization, indicating that cathepsins, unlike human neutrophil elastase, are not suitable markers of *P. aeruginosa* infection.

## Introduction

Cystic fibrosis is an inherited life-threatening disorder. It is associated with a mutation of the CF transmembrane glycoprotein that is involved in the transport of chloride ions [1], [2]. This exocrinopathy mainly affects cells producing mucus, sweat and digestive fluids and causes severe lung damage and nutritional deficiencies. While palliative care is presently available for these patients, there is no effective cure [3]. The clinical manifestations of chronic inflammation of the respiratory epithelium (overproduction of mucus, persistent cough, wheezing, repeated lung and sinus infections), are mainly due to the release of proteolytic enzymes and the disruption of the protease-antiprotease balance. This leads to the degradation of lung tissues and the impairment of lung function. The involvement of serine proteases released from polymorphonuclear neutrophils (elastase, cathepsin G, protease 3) has been extensively studied [4], [5] but the role of lung cysteine proteases (CPs, family C1) is less well documented [6]. The cysteine cathepsins B, H, L, K and S are involved in a variety of proteolytic processes, such as the turnover of endocytosed proteins, prohormone processing, MHC-II antigen presentation, and extracellular matrix and basal membrane degradation. They are also involved in diseases like tumor metastasis, osteoporosis, and rheumatoid arthritis [7], [8]. Lung CPs are mainly produced by macrophages, fibroblasts and epithelial cells, while cathepsin H is mainly found in type II pneumocytes [9], [10]. Stimulated monocyte-derived macrophages can release the CPs that are found in the bronchoalveolar lavage fluids (BALFs) of smokers suffering from emphysema [11]. Active forms of cysteine cathepsins are also present in BAL fluids from patients suffering from infiltrative inflammatory disorders like sarcoidosis and alveolar proteinosis, and silicosis [12], [13], [14].

The hyperviscous mucus found in CF airways severely hinders effective phagocytosis by neutrophils and makes the lungs more susceptible to infection by *Pseudomonas aeruginosa*, *Staphylococcus aureus*, and *Haemophilus influenzae*. While *S. aureus* is predominantly found in the early stages of colonization, *P. aeruginosa* is more resistant to antibiotics and soon becomes the main organism infecting CF lungs [15]. Cysteine cathepsins may be important in the pathophysiology of cystic fibrosis under these conditions [16]. The activity of cathepsin B is dramatically higher (∼several 100-fold) in the bronchoalveolar lavage fluids of CF patients than in those of healthy patients [17]. This high proteolytic activity in CF lungs may contribute to the dysfunction of the inflammatory response and thus to local tissue damage [16]. Cathepsins may also exacerbate lung disease by weakening the host defenses by breaking down and inactivating SLPI (secretory leukocyte protease inhibitor), beta-defensins 2 and 3 (HBD-2 and HBD-3), and lactoferrin. The resulting loss of antimicrobial activity favors infection and colonization by opportunistic pathogens [17], [18], [19]. The cathepsin activity in lavage fluid and sputum from CF patients whose lungs are colonized by *P. aeruginosa* is higher than reported for healthy controls [18]. A recent study detected both cathepsins B and S in CF sputum samples and proposed their use as markers of CF airway inflammation. The authors also suggested that the concentrations of both enzyme were correlated not only with each other but also with those of neutrophil elastase and IL-8 [20].

However, changes in the concentration and activity of CPs must be carefully examined before it can be stated that CPs may be markers of inflammation and/or bacterial infection and colonization [21]. Thus, the primary purpose of this study was to describe the enzymatically active forms of cysteine cathepsins in *P. aeruginosa*-positive (Ps+) and *P. aeruginosa-*negative (Ps−) CF expectorations using a single protocol. We then estimated the implications of the imbalance between CPs and their specific circulating inhibitors (kininogens, cystatins) by quantitative kinetic analysis based on protein content rather than sputum volume (or weight), as reported elsewhere. Finally, we analysed these data to determine whether CP activities were correlated with *P. aeruginosa* colonization and whether CPs may be useful new biological markers.

## Materials and Methods

### Substrates and synthetic inhibitors

Benzyloxycarbonyl-Arg-Arg-7-amino-4-methyl coumarin (Z-Arg-Arg-AMC), H-Arg-AMC, Z-Gly-Pro-Arg-AMC and Z-Phe-Arg-AMC were purchased from Bachem (Weil am Rhein, Germany) and Z-Val-Leu-Arg-AMC from Enzyme System Products (Livermore, CA, USA), ortho-aminobenzoic acid (Abz)-Ala-Pro-Glu-Glu-Ile-Met-Arg-Arg-Gln-(3-NO_2_-Tyrosine) came from GeneCust Europe (Dudelange, Luxembourg). L-3-carboxy-trans-2, 3-epoxy-propionyl-leucylamide-(4-guanido)-butane (E-64), PMSF, pepstatin A, EDTA, 4-(2-Aminoethyl) benzenesulfonyl fluoride hydrochloride (AEBSF; Pefabloc) and MMTS were from Sigma-Aldrich (Saint-Quentin Fallavier, France). N-(4-Biphenylacetyl)-S-methylcysteine-(D)-Arg-Phe-β-phenethylamide and N-(L-3-trans-propylcarbamoyloxirane-2-carbonyl)-L-isoleucyl-L-proline (CA-074) were from Calbiochem (VWR International S.A.S., France). Morpholine urea-Leu- homophenylalanine-(vinylsulfonyl)benzene (Mu-Leu-Hph-VSPh) was kindly provided by Dr J.H. McKerrow (Department of Pathology, The Sandler Center for Basic Research in Parasitic Diseases, University of California, San Francisco, CA, USA). The biotinylated activity-based probe Biot-LVG-CHN_2_ was synthesized as previously described [22]. DTT (DL-dithiotreitol) came from Bachem. All other reagents were of analytical grade.

### Enzymes and inhibitors

Human cathepsins B, H, L and S were supplied by Calbiochem and human neutrophil elastase by BioCentrum (Krakow, Poland). High molecular weight kininogen (HMWK) was purchased from Calbiochem and cystatin C from R&D Systems Europe.

### Ethics Statement

Sputum samples were collected on a routine basis from adult patients followed at the Teaching Hospital of Besançon (CHU Jean Minjoz, France) between 2009 and 2010. Enzymatic assays were performed in addition to routine bacteriological analyses when the volume of sputums was sufficient for both types of tests. Thus our protocol was considered as “waste” and we did not need a specific agreement from the local research ethics committee.

### CF sputum samples

Twenty five sputum samples were collected (status reported as means ± S.D: years, 27.1(9.4); forced expiratory volume (FEV), 2.0 (0.9) L; body mass index (BMI), 20.4 (2.8)). Very soon after their recovery, the specimens were aseptically divided in two parts. One half was submitted to conventional bacteriological analyses to identify and quantify (colony-forming units per mL, cfu/mL) bacterial pathogens [23]. Sensitivity of detection of *P. aeruginosa* was ≥20 cfu/mL. Nineteen *P. aeruginosa*-positive samples and six *P. aeruginosa*-negative samples used as controls were included in the study. After thawing of the other half and to allow the accurate handling of clinical specimens, a preservative buffer (final concentrations: 100 mM sodium acetate, pH 5.0 plus the peptidase inhibitors 0.5 mM PMSF, 0.5 mM EDTA, 40 µM pepstatin A, and 1 mM MMTS) was instantly added to each CF sputum sample before it was centrifuged at 5000 g at 4°C for 10 min. The resulting cell-free supernatants were collected, aliquoted and frozen at −80°C. Alternatively a second buffer, 50 mM HEPES pH 7.4, 150 mM NaCl, 0.05% NP40, 0.5 mM EDTA, 40 µM pepstatin A, and 1 mM E-64, was used for further analysis of elastase activity.

### Immunoblotting

The goat anti-human cathepsin S was obtained from R&D Systems. Other primary polyclonal antibodies were raised in rabbits: anti-human cathepsin B (Calbiochem), anti-human cathepsin L (Calbiochem), anti-human cathepsin H (Fitzgerald, Concord, USA), anti-human cystatin C (Upstate, Lake Placid, USA) and anti-human low molecular weight kininogen [24]. The anti-human neutrophil elastase (HNE) was raised in rabbits using a 16-mer peptide corresponding to position 88–103 (IFENGYDPVNLLNDIV) of the proelastase sequence (numbering based on the sequence of prochymotrypsinogen [5]) coupled to ovalbumin for immunization. The IgG fraction obtained after ammonium sulfate precipitation was further purified by affinity chromatography on immobilized ovalbumin. Goat anti-rabbit and rabbit anti-goat IgG-peroxidase conjugates were supplied by Sigma-Aldrich. Bicinchoninic acid assays were used to determine the protein concentrations in supernatants (BCA protein assay kit, Interchim, Montluçon, France). Samples (30 µg protein) were diluted in Laemmli buffer under reducing conditions, boiled for 5 min, separated by SDS-PAGE on 15% gels (prestained molecular masses: Precision Plus Protein Standards, BioRad) and electroblotted onto nitrocellulose membranes. These membranes were incubated with the primary antibody (1∶1000, in PBS, 0.1% Tween, 5% dried milk for 1 h at room temperature), then with the secondary IgG-peroxidase conjugate (1∶5000) for 1 h at room temperature. Proteins were detected by chemiluminescence (ECL Plus Western Blotting Detection system, Amersham Biosciences, Buckinghamshire, UK). This protocol was used for *P. aeruginosa*-positive (Ps+) and *P. aeruginosa*-negative (Ps−) samples.

### Labeling cysteine cathepsins with a cystatin-derived activity-based probe

Supernatants in buffer A (100 mM sodium acetate buffer pH 5.5 containing 5 mM DTT, 2 mM EDTA and 0.01% Brij 35) were incubated with a molar excess of Biot-LVG-CHN_2_ (cathepsin∶probe, 1∶300) for 1 h at 37°C, as described previously [25]. In another set of experiments, we first incubated samples with unlabelled inhibitors (100 µM): E-64 (a broad-spectrum cathepsin inhibitor), CA-074 (a selective cathepsin B inhibitor) or Mu-Leu-Hph-VSPh (a selective cathepsin S inhibitor) before adding the biotinylated probe. Individual cathepsins B, H, L and S were also used as control. Samples were then separated by SDS-PAGE on 12% gels under reducing conditions and transferred to nitrocellulose membranes by electroblotting. Free binding sites on the membranes were saturated by incubation with 3% BSA in PBS for 1 h at 37°C. The membranes were incubated with an extravidin-peroxidase conjugate (1∶2500; Sigma-Aldrich) for 2 h at room temperature and the peroxidase activity was revealed by chemiluminescence (ECL Plus Western Blotting Detection system).

### Hydrolysis of kininogens by CF sputum

Human HMWK (1.8 µg) was incubated in 100 mM sodium acetate buffer pH 5.5, 5 mM DTT, 2 mM EDTA, 0.01% Brij 35 with supernatant (corresponding to 10 µg protein) at 30°C for 5 hours. The mixture was then separated by 12.5% SDS-PAGE under reducing conditions and the separated products transferred to nitrocellulose membranes. Control mixtures contained E-64 and CA-074. HMWK hydrolysis was detected using a rabbit polyclonal anti-kininogen antibody [24].

### Enzyme activity

A panel of AMC-derived fluorogenic substrates was used to measure the CP activities in supernatants diluted in buffer A. Samples (final volume per well: 200 µL) in 96-well Nunc microtiter plates (ThermoFisher Scientific, Illkirch, France) were incubated at 37°C under gentle agitation, and their enzymatic activities were monitored continuously at λ_exc_ = 350 nm and λ_em_ = 460 nm (Gemini spectrofluorimeter, Molecular Devices, Saint-Grégoire, France). The protocol for quantifying active cathepsins in sputum supernatant was adapted from that used to titrate the CPs in BAL fluids [12], [13]. Supernatant was incubated with concentrations of E-64 (0–100 nM) at 37°C for 30 min [26] in buffer A and the residual endopeptidase CP activity towards Z-Phe-Arg-AMC (20 µM) was then measured. Cathepsin B was titrated with CA-074 (0–100 nM) using Z-Arg-Arg-AMC as substrate (5 µM). Certain samples were incubated with CA-074 to inhibit cathepsin B before cathepsin K was titrated with E-64 using Z-Gly-Pro-Arg-AMC as substrate (50 µM), and cathepsin S using Z-Val-Leu-Arg-AMC as substrate (20 µM). The concentration of cathepsin L was deduced from the difference between the overall concentration of thiol-dependent endoproteases (i.e cathepsins B+K+L+S) and the individual concentrations of cathepsins B, K and S. The aminopeptidase activity of cathepsin H was assayed using H-Arg-AMC (50 µM) as substrate. As cathepsin S is more stable than the other CPs at neutral pH, its specific activity was also assayed under mildly alkaline conditions [27]. Supernatant was incubated in 100 mM Na-phosphate buffer pH 7.4 (80 µL) for 1 hour at 37°C. An aliquot was then removed, diluted with buffer A and used to measure the residual cathepsin S activity at 37°C with Z-Val-Leu-Arg-AMC (20 µM) as the substrate. HNE activity was measured in the supernatant (1∶50) using Abz-Ala-Pro-Glu-Glu-Ile-Met-Arg-Arg-Gln-(3-NO_2_-Tyr) (10 µM) in buffer B ( 50 mM HEPES buffer pH 7.4, 150 mM NaCl, 0.05% NP40).

### Inhibitory potential of CF sputum

The procedure used to evaluate the inhibitory potential of supernatants was adapted from Assfalg-Machleidt et al. [28]. As most of the thiol-dependent endoprotease activities in sputum are those of cathepsins B and L (about 80%), their CP activities were blocked by incubating samples for 30 minutes with 0.2 µM CA-074 and 2 µM N-(4-Biphenylacetyl)-S-methylcysteine-(D)-Arg-Phe-β-phenethylamide. The inhibitory potential of CF sputum was deduced by adding increasing amounts of sputum supernatants (0–14 µL) to E-64 titrated papain, using Z-Phe-Arg-AMC as substrate (5 µM).

### Zymogen activation in CF sputum

Diluted supernatants (20 µL, corresponding to 20 µg protein) were incubated in 800 µL 100 mM sodium acetate buffer pH 4.3, 4 mM DTT, 10 µg/ml dextran sulfate (Sigma-Aldrich), or buffer B at 37°C. Aliquots (2 µL) were removed at intervals (0–6 hours) and the CP activity in them was measured at 37°C, in buffer A, using Z-Phe-Arg-AMC (50 µM). E-64 was used as control. Samples were analyzed in parallel by Western blotting, as described above. Alternatively aliquots of supernatant were incubated in 50 mM HEPES (pH 7.4), 150 mM NaCl, 0.05% NP40 at 37°C to assay the elastase-dependent maturation of cathepsin B, using AEBSF (Pefabloc) as control.

### Statistical analyses

Results were analyzed with the non-parametric Mann–Whitney U test; a P value<0.05 is considered to be statistically significant.

## Results and Discussion

### Immunodetection and labeling of cysteine cathepsins and their inhibitors

**Figure 1:**
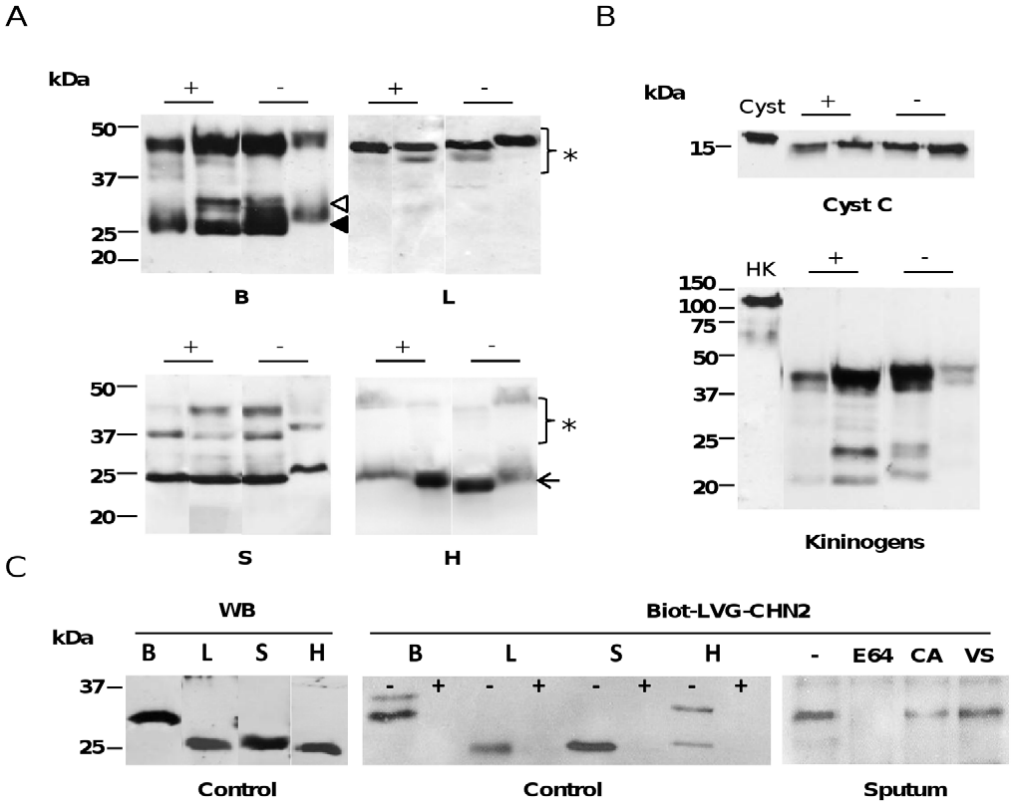
Cysteine cathepsins and their inhibitors in supernatants of CF sputum. Only representative samples are shown. (A) Proteins (30 µg/well) were separated by 15% SDS-PAGE under reducing conditions, transferred to nitrocellulose membranes, and analyzed with polyclonal antibodies against human cathepsins B, H, L and S. (+): *Pseudomonas aeruginosa*-colonized CF sputum; (−): *Pseudomonas aeruginosa*-negative CF sputum.◃, single-chain cathepsin B; ◂, double-chain cathepsin B; ←, mature cathepsins S and H; ★, proforms. (B) Immunostaining with polyclonal anti-cystatin C antibody and anti-kininogen antibody. (+): *Pseudomonas aeruginosa*-colonized CF sputum; (−): *Pseudomonas aeruginosa*-negative CF sputum. Control: Cyst, Cystatin C; HK, HMWK. Recombinant human cystatin C (R&D systems) has an additional C-terminal 10 His-tag and an apparent molecular mass of 17 kDa, according to the supplier. (C) Supernatants of CF sputum incubated with Biot-LVG-CHN_2_ (30 µM), for 1 h at 37°C [25]. Other samples were pre-incubated with E-64, CA-074, and Mu-Leu-Hph-VSPh prior to adding the biotinylated activity-based probe. Samples were separated by 12% SDS-PAGE, electroblotted and incubated with extravidin-peroxidase conjugate. The peroxidase activity was revealed by chemiluminescence. WB: individual cathepsins B, H, L and S immunoblotted as control. Control: (−), no pre-incubation with E-64; (+), pre-incubation with E-64 prior to adding Biot-LVG-CHN2. Sputum: E-64, pre-incubation with E-64; CA, pre-incubation with CA-074; VS, pre-incubation with Mu-Leu-HphVSPh.

The supernatants obtained by centrifuging the 25 *Pseudomonas aeruginosa*-positive and *Pseudomonas aeruginosa*-negative CF sputum samples were immediately buffered at pH 5.5 and stabilized (see the experimental section) to preserve cysteine cathepsins from inactivation at neutral pH and uncontrolled proteolysis. The median protein concentration was 3.74 mg/ml with interquartile ranges: IQR1, 3.31 mg/ml and IQR3, 4.50 mg/ml. We detected aminopeptidase cathepsin H in all sputum supernatants in addition to cathepsins B, L and S reported by Taggart et al. [17] (see representative samples in Figure 1A). However the activity profiles varied considerably, depending on the cysteine protease studied. Cathepsin L was mostly in its proform; the concentration of its mature form was below the limits of immunodetection under our experimental conditions. The full and/or partly processed proforms of cathepsin K were also detected, but mature cathepsin K was not (data not shown; the anti-cathepsin K antibody was a kind gift from Dr Dieter Brömme, University of British Columbia, Vancouver, Canada). Conversely, cathepsin H was detected mainly as its mature form. Both mature cathepsin S and procathepsin S were found (full or partially processed proform, depending on the sample). Mature cathepsin B was intensely stained (mainly as its double-chain form). But the zymogen of this most abundant and ubiquitous cathepsin was also found. However, Martin et al. [20] did not report finding pro-cathepsin B. This discrepancy could be due to dissimilar storage conditions, since they diluted their expectorated sputum with an unbuffered saline without adequate inhibitors to stabilize them. There were small differences in the apparent molecular weights in sputum CPs and controls that reflect their degree of glycosylation. The enzyme/proenzyme profiles of sputum samples differed from those of BAL fluids from patients suffering from infiltrative inflammatory disorders [13], where cathepsins H and L were mostly detected as mature forms and cathepsins B, K and S as proforms. The enzyme profiles also differed from those of BAL fluids from silicosis patients, where only the mature forms of cathepsins B, H and L were immunodetected, while cathepsin H was the most abundant CP [12]. However, the immunochemical patterns of *P. aeruginosa*-positive and *P. aeruginosa*-negative samples were fairly similar for cathepsins and their endogenous inhibitors.

Cystatin C and kininogens, the major plasma circulating inhibitors of cathepsins, have been also found in some inflammatory bronchoalveolar lavage fluids and epithelial lining fluids, but never before in CF samples (see for review: [6]). Our Western blot analysis detected cystatin C and high and/or low molecular weight kininogens in CF sputum (Figure 1B). Despite sputum HNE was reported to cleave cystatin C and release a N-terminally truncated form [29], we did not observe the presence of a shorter molecular form of cystatin C, as also suggested by agarose electrophoresis (data not shown). Conversely no intact but extensively degraded kininogens were detected. Similar degradation of kininogens have been found in other inflammatory body fluids, like synovial and amniotic fluids, and blood plasma (for review: [30]). The lack of undamaged kininogens together with the presence of kininogen fragments (*circa* 20–40 kDa) correlates with the recent demonstration that the inability of kininogens to inhibit cathepsin B (in contrast to cathepsins L and S) is associated with their extensive cleavage by cathepsin B [31]. We have also shown that Biot-LVG-CHN_2_, a cystatin C-derived activity-based probe, binds to sputum cysteine cathepsins. Biot-LVG-CHN_2_ efficiently labeled human cathepsins B, H, L and S (Figure 1C) by specifically targeting the nucleophilic active site thiol. One major band was found in CF sputum both with and without a *Pseudomonas* infection. This labeling was abolished by E-64, broadly impaired by preincubation with CA-074, and to a lesser extent by Mu-Leu-Hph-VSPh. This provides strong evidence that cathepsin B (predominant reactive band corresponding to its double-chain form) is the most abundant CP, and that active cathepsin S is also present in CF sputum. Specific labeling with Biot-LVG-CHN_2_ also confirmed that CPs, despite their susceptibility to thiol oxidation and the partially defective antioxidant defenses in the lung, may retain their enzymatic activity for some time in an oxidative environment [32], [33], [34].

**Figure 2:**
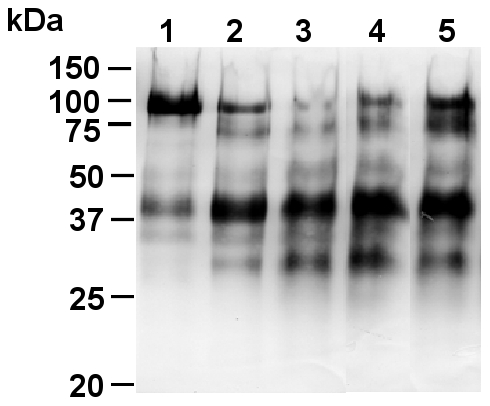
Degradation of human HMWK by CF sputum. Exogenous HMWK was incubated in the activity buffer with supernatants of CF sputum at 30°C for 0–5 hours. Hydrolysis products were separated by 12.5% SDS-PAGE, transferred to nitrocellulose membranes and immunoblotted with rabbit polyclonal anti-kininogen antibody [24]. Lane 1, 0-h incubation; lane 2, 2-h incubation; lane 3, 5-h incubation; lane 4, 5-h incubation in the presence of CA-074; lane 5, 5-h incubation in the presence of E-64. For clarity, one representative sample is shown.

As both high and low molecular weight kininogens were broken down in both Ps+ and Ps− CF sputum (Figure 1B), we added exogenous uncleaved HMWK to CF supernatants and analyzed the resulting mixture. E-64 and also CA-074 partially blocked the cleavage of exogenous HMWK (Figure 2), indicating that cathepsins play a part in the proteolysis of kininogens by CF sputum. Our data also confirmed that significant amounts of cathepsins may escape regulation by their endogenous inhibitors. This, together with the recent demonstration that the poor inactivation of cathepsin B (unlike the tight-binding inhibition of cathepsins L and S) by kininogens is associated with their extensive cleavage by cathepsin B [31], indicates that cathepsin B is a major protease involved in this process in CF sputum. This is also true for several other inflammatory disorders (for review: [30]).

### Quantitative analysis of sputum cysteine cathepsins and their inhibitors

**Figure 3:**
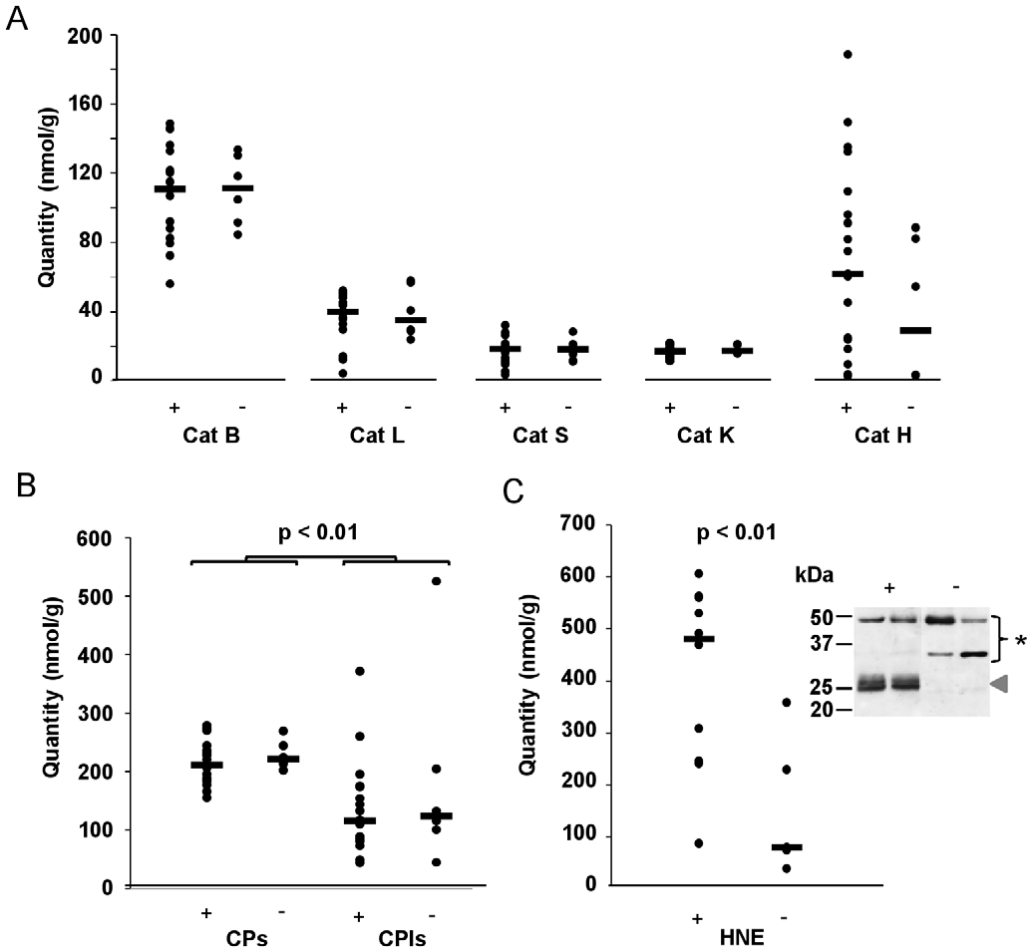
CP and HNE activities, and CP/CPI balance in CF sputum. (+): *P. aeruginosa*-colonized CF sputum; (−): *P. aeruginosa*-negative CF sputum. Cathepsins B, H, K, L and S and HNE activities were quantified as reported in details in the experimental section. Data are shown as individual points and statistically significant P values are shown. The horizontal bars indicate medians. (A) Cathepsins B, L, S, K and H. (B) CP/CPI balance. CPI: expressed as inhibitory site (cystatin-like) equivalent. (C) Elastase activities: the horizontal bars indicate medians. The western blot analysis was performed using a rabbit anti-HNE antibody (representative samples are shown). ◂, unbound HNE; ★, bound HNE.

The characteristics of active proteases are expressed with reference to the total protein concentration in CF samples and not to volume (or dilution) of sputum supernatants in order to prevent any bias associated with clinical specimen during sputum collection. The BCA assays indicated that the median protein concentration in the 25 samples tested was 3.74 mg/ml (IQR1, 3.31 mg/ml; IQR3, 4.50 mg/ml; range, 1.21–14.25 mg/ml). All concentrations of active enzymes and inhibitors are given as medians, with IQR1 and IQR3 values in brackets. We first determined the specific activity of human neutrophil elastase using the FRET substrate Abz-Ala-Pro-Glu-Glu-Ile-Met-Arg-Arg-Gln-(3-NO_2_-Tyr) whose amino acid sequence is highly selective for HNE [35]. The median values of active HNE in *P. aeruginosa*-positive samples were 480.4 (261.5/552.4) nmol/g protein and 80.4 (74.9/229.4) nmol/g in *P. aeruginosa*-negative samples. Thus, HNE activity in Ps+ CF sputum was significantly higher than in the Ps-CF sputum (p-value<0.01) (Figure 3C). The increased sputum HNE activity that is typically associated with a bacterial infection (mainly *S. aureus* and *P. aeruginosa*) and the ensuing influx of neutrophils agrees well with earlier reports indicating that elastase is a valuable marker of infection for CF patients (see for review: [36]). This is supported by the results of immunoblotting following SDS-PAGE under reducing conditions. A polyclonal anti-HNE antibody revealed a major band corresponding to free unbound elastase in Ps+ CF sputum (Figure 3C), while HNE in Ps− CF samples was mostly as higher molecular forms that correspond most probably to inhibitory complexes between HNE and alpha1-protease inhibitor (α1-PI), the major serine protease inhibitor in the lung [5]. Targeting of HNE by α1-PI obey the suicide substrate inhibition mechanism of serpins with cleavage within the reactive center loop of α1-PI and the formation of a 1∶1 stoichiometric covalent inhibitory complex [37], [38], [39]. The individual (Figure 3A) and overall (Figure 3B) concentrations of active CF cathepsins were determined by titration (see “Material & Methods” for details). The overall active cathepsins in *P. aeruginosa*-positive samples was 211.5 (187.5/234.2) nmol/g, while the activity in *P. aeruginosa*-negative samples was 221.7 (214.8/239.6) nmol/g. This value is higher than that measured in silicosis BALFs, but lower than the concentration of active CPs in BALFs from patients with acute lung infiltrative inflammatory disorders [6]. Cathepsin B is the most abundant cathepsin in CF sputum (Ps+ median: 111.5 (90.4/127.9) nmol/g; Ps− median: 112.1 (95.4/127.8) nmol/g), as suggested by immunochemical studies, followed by the aminopeptidase cathepsin H (Ps+ median: 62.4 (21.7/103.3) nmol/g; Ps− median: 29.5 (3.8/75.8) nmol/g) and the endopeptidase cathepsin L (Ps+ median: 40.5 (31.8/47.4) nmol/g; Ps− median: 35.6 (29.7/53.3) nmol/g). The median concentrations of cathepsin S were 19.1 (12.2/21.6) nmol/g in Ps+ sputum and 18.8 (13.3/21.6) nmol/g in Ps− CF sputum; the values for cathepsin K were similar: 17.7 (15.4/18.9) nmol/g in Ps+ sputum and 17.7 (17.2/20.6) nmol/g in Ps− CF sputum. The differences between *P. aeruginosa*-positive and *P. aeruginosa*-negative samples were not significant (p>0.05), including the apparent variation in cathepsin H. We also found no correlation between the elastase and cathepsin B activities, in contrast to the findings for sputum from patients with bronchiectasis [29].

We assessed the residual CP inhibitory capacity (CPI, expressed as inhibitory site equivalents) by measuring the ability of CF sputum to inhibit E-64-titrated papain, according to Assfalg-Machleidt et al. [28]. Again, we found no significant difference between *P. aeruginosa*-positive (116 (83.8/168.2) nmol/g) and negative (123.9 (104.7/186.6) nmol/g) samples (Figure 3B), showing no significant difference between the two groups. Conversely, the CP/CPI imbalance (∼2-fold) was statistically significant (p<0.001) in both cases. Taken together that cleavage of cystatin C by HNE was known to lead to a critically weaker inhibition (three orders of magnitude) of cathepsin B [29], and that the CP/CPI imbalance is unchanged in both Ps+ and Ps− groups, results also support that cystatin C may be protected from the harmful activity of HNE in CF expectorations. On the other hand the CP/CPI imbalance is smaller than that in BALF from patients with acute infiltrative inflammatory disorders (CP/CPI balance: 3- to 5-fold), due to the concentration of active CPs being lower in CF sputum. These data indicated that cathepsin activities are out of control in chronic inflammation disorders, which supports the hypothesis that CPs take part in the degradation and remodeling of major ECM and BM components that are associated with the progression of the disease. But the exact contributions of individual cathepsins to the pathophysiology of CF remain unclear [16].

However our use of a standardized procedure in which proteolytic activities are expressed with reference to the protein content of each sample (nmole/g) confirms that HNE is a valuable marker of infection by *P. aeruginosa* (P<0.01), regardless of the method used. Our data also indicate that cathepsin activities may not be used as a reliable indicator of bacterial colonization and *Pseudomonas* infection, unlike a previous proposal [18]. Although it is well established that increased clearance promotes an influx of cells, which may increase the protein concentration in the sputum, these contradictory results underline that our qualitative analysis are not expressed with reference to sputum sample volume (or weight), which can vary during specimen collection.

### Zymogen activation

**Figure 4:**
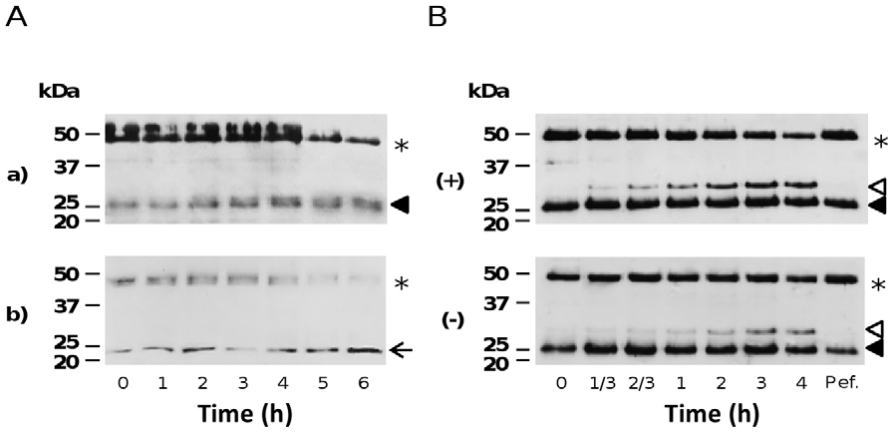
Maturation of human procathepsins in CF sputum. (A) Autocatalytic maturation of procathepsins B and S. Supernatants were incubated for up to 6 h in 100 mM sodium acetate buffer pH 4.3, 10 µg/ml dextran sulfate, 4 mM DTT, at 37°C. The maturation products were separated by 12% SDS/PAGE under reducing conditions, transferred to a nitrocellulose membrane and analyzed by western blotting using polyclonal antibodies specific for (a) human cathepsin B and (b) human cathepsin S. One representative sample is shown. ★, proforms; ◂, mature double-chain cathepsin B; ←, mature cathepsin S. (B) Elastase-dependent maturation of procathepsin B. Supernatants were incubated for up to 4 h in 50 mM HEPES pH 7.4, 150 mM NaCl, 0.05% NP40 at 37°C and the maturation products analyzed by immunoblotting using a polyclonal anti-cathepsin B antibody. Pefabloc was used as control (4 h). Pef, Pefabloc (AEBSF). (+): *P. aeruginosa*-positive CF sputum (one representative sample); (−): *P. aeruginosa*-negative CF sputum (one representative sample). ◃, single-chain cathepsin B; ◂, double-chain cathepsin B; ★, procathepsin B.

High-Mr forms of extracellular cathepsin B, corresponding to a stable, noncovalent complex between cathepsin B and its 6-kDa propeptide, have been found in the media of mammary tumor explants and in purulent sputum from patients with bronchiectasis, an obstructive lung disease like emphysema and cystic fibrosis, with impaired clearance of mucous secretions [40]. Breakdown of the inhibitory propeptide resulted in increased enzymatic activity, indicating that extracellular stabilized cathepsin B can be dormant. We detected no such complexes in CF sputum, but we did find substantial extracellular amounts of immunoreactive procathepsins B, L and S (Figure 1). Thus the presence of acid oligosaccharides at cell surfaces [41] and the local acidic micro-environnements in CF epithelial lining fluids [16] should lead to weaker interactions between the proregion and the catalytic domain, which may favor the autoproteolytic conversion to mature active cathepsins (for review: [42]). We investigated the *in vitro* activation of procathepsins B and S by incubating CF sputum under acidic conditions (Figure 4A). The two procathepsins had similar maturation patterns, and both were abolished by E-64 (data not shown). The band corresponding to the mature protease intensified and became maximal at 5 hours. The autocatalytic processing of cathepsin B led to the release of the ∼24/25 kDa (double-chain), but not the ∼31 kDa form, as mainly observed in CF sputum (Figure 1). This was associated with a relatively small (∼1.6-fold) increase in proteolytic activity against AMC peptides, due to the concomitant time-dependent inactivation of cysteine cathepsins at 37°C. The quiescent procathepsins in CF sputum correspond to an activateable proteolytic reserve that may strengthen the CP/CPI imbalance and promote the deleterious elastinolytic and collagenolytic activities of cathepsins, especially during exacerbation episodes. This is in contrast to the situation in samples from patients with silicosis [6]. Incubation of CF sputum at a weakly basic pH also led to the proteolytic release of cathepsin B (∼31 kDa; the single-chain form). Immunoblotting also showed that the rate and yield of activation were much greater in Ps+ sputum than in Ps− CF sputum (Figure 4B), while the processing of procathepsin B was impaired by Pefabloc, an irreversible serine protease inhibitor. Taken together that HNE activity in Ps+ CF sputum was significantly higher than in the Ps-CF sputum and a former report by Buttle et al. [43], the present observation supports that HNE may possibly process procathepsin B to its active form. However, our data also suggest that a such serine protease-dependent maturation of cathepsin B probably does not occur primarily in either *P. aeruginosa*-positive or *P. aeruginosa-*negative CF sputum, since the major band detected by immunoblotting and active-site labeling corresponded to the double-chain cathepsin B.

In conclusion, we identified active cathepsins B, H, K, L and S in sputum from CF patients, as well as proforms that may be processed autocatalytically or possibly by elastase (cathepsin B), in agreement with the overproduction and secretion of cysteine cathepsins in chronic lung inflammatory diseases [44]. We also found that kininogens, their natural circulating inhibitors, are extensively degraded. Thus the overall imbalance between cysteine proteases and related inhibitors favors the uncontrolled proteolytic activities of cathepsins. These uncontrolled cathepsins could then contribute to the pathophysiological breakdown/remodeling of the extra cellular matrix components that occurs in cystic fibrosis. Cathepsins also seem play a critical role in the regulation of the antimicrobial activity of innate immunity proteins in cystic fibrosis, thus favoring the colonization by pathogens like *P. aeruginosa*, and infection. Cysteine cathepsins cleave beta-defensins, lactoferrin, and secretory leukocyte protease inhibitor and abrogate their microbicidal activity [44]. However we observed no significant difference in the CP activities and CP/CPI imbalance of *P. aeruginosa*-positive samples and *P. aeruginosa*-negative samples. Hence the cathepsin activities cannot be used as an indicator of colonization by this pathogen in CF patients. Unlike neutrophil elastase, human cathepsins are not consistent markers of infection by *P. aeruginosa* in CF patients.
